# Prevalence of sleep disturbances and their association with quality of life among the general population in Qassim, Saudi Arabia

**DOI:** 10.1371/journal.pone.0347188

**Published:** 2026-08-06

**Authors:** Jolan Alsaud, Ghadi Alkhalaf, Rand Alsaleem, Betool Alqefari, Haifa Alfozan, Lama Alqaraawi, Siraj Wali

**Affiliations:** 1 Qassim Health Cluster, Qassim, Saudi Arabia; 2 Patients’ Friends Association, Unayzah Governorate, Qassim, Saudi Arabia; 3 King Abdulaziz University, Jeddah, Saudi Arabia; University of Rijeka Faculty of Health Studies: Sveuciliste u Rijeci Fakultet zdravstvenih studija, CROATIA

## Abstract

To the best of our knowledge, no previous study has assessed the relationship between sleep disturbances and quality of life in the general population of Qassim, Saudi Arabia. Therefore, this cross-sectional study aimed to evaluate the relationship between sleep disorders and quality of life and to determine how common sleep disturbances are in Qassim, Saudi Arabia. To this end, participants were randomly selected from public areas. Study data were collected through a survey covering sociodemographic details, health information, the Pittsburgh Sleep Quality Index (PSQI) to assess sleep quality, and the Short Form-12 to measure health-related quality of life. The reliability of the scales was confirmed using Cronbach’s alpha. The association between sleep quality and quality of life was analyzed using t-tests, Pearson’s correlation, and hierarchical multiple regression. Of 1,241 participants, 64.5% were females, 94.8% were Saudi nationals, and 64.8% were students. The mean global PSQI score was 7.38 ± 3.19, with 70.2% of participants reporting poor sleep quality. The mean physical and mental component scores were 42.6 ± 7.98 and 38.9 ± 8.03, respectively. Hierarchical multiple regression analyses indicated that after controlling for demographic characteristics, chronic disease, and body mass index, sleep-related variables significantly predicted both physical and mental health-related quality of life. The analysis showed that sleep-related factors had a substantially larger predictive contribution toward mental health (ΔR^2^ = 5.7%) than toward physical health (ΔR^2^ = 1.0%). In conclusion, this study revealed a high rate of poor sleep quality (70.2%) in Qassim, Saudi Arabia, with notable negative effects on mental health. Increasing awareness and integrating sleep assessments into public health initiatives are crucial for improving sleep quality.

## Introduction

Generally, estimates of the prevalence of sleep disturbances depend on the criteria used to define sleep disturbances and the population being studied. Approximately 30% of the adult populations of different countries reported one or more symptoms of sleep disturbances, such as difficulty in maintaining or initiating sleep, waking up too early, and in some cases, non-restorative or poor sleep quality [1]. Furthermore, a study of more than 40,000 senior citizens from eight nations in Asia and Africa revealed an increase in sleep issues [2]. The prevalence of sleep disruptions among Saudi adults has been estimated to be 33.8%, with the incidence in women being higher than that in men (37.3% vs. 31.4%) [3]. Lower quality of life, functional impairment, and a higher risk of suicidal behavior are all associated with sleep disturbances. Additionally, evidence suggests that sleep issues may influence the development of depression [4].

Sleep impairment, insomnia, and other sleep disorders influence physical, emotional, and mental well-being, thus affecting quality of life [5]. In 2019, a cross-sectional study conducted in Lebanon used the 12-item Short Form (SF-12) Health Survey and found that a family history of insomnia can strongly influence physical quality of life. Additionally, the mental quality of life declines with increasing age [6]. A study on insomnia in children conducted in Hong Kong showed a strong familial connection between children’s biological parents and insomnia in children, even after adjusting for sociodemographic factors [7].

A study on adolescent quality of life showed that good sleepers had higher health-related quality of life (HRQoL) than those with poor sleep patterns. Nonetheless, the study showed that sex influences sleep quality: The prevalence of sleep disorder was higher in girls than in boys, which affected their quality of life [8]. Another study showed that insomnia prevalence in older adults was 62.1%, and was much higher in those with other health issues such as depression and fear of death [9]. Adolescents appear to be particularly vulnerable to sleep disturbances. In a study involving 855 high school students aged between 14 and 19 years, the average sleep duration was only 7 h and 14 min ± 1 h and 20 min, significantly below recommended levels. Additionally, one study identified a correlation among shorter sleep duration, lower quality of life, and increased depression [10].

Several physical, psychological, and social factors affect sleep quality. Serap et al. conducted a study among university students to identify factors affecting sleep quality. Sleep quality was found to be influenced by the students’ financial status, place of residence, number of room occupants, routine bedtime habits, problems during sleep, how rested they felt upon waking up in the morning, family history of sleeping problems, and frequent smoking and alcohol consumption [11]. An additional study was conducted to determine factors influencing college students’ sleep quality during the COVID-19 pandemic. The results showed that sleep quality was significantly affected by intolerance of uncertainty and anxiety about COVID-19, suggesting that mental health can also affect sleep quality [12].

To the best of our knowledge, no previous study has assessed the relationship between sleep disturbance and quality of life in the general population in Qassim, Saudi Arabia. Therefore, we aimed to fill this gap by determining the prevalence of sleep disturbances in the general population of the Qassim region of Saudi Arabia and to assess the relationship between sleep disturbances and quality of life.

## Materials and methods

This cross-sectional study was conducted in the Qassim, Saudi Arabia, between October 1, 2023, and February 28, 2024. Qassim. an administrative province in Saudi Arabia, has an area of 65,000 km² and a population of approximately 1,016,756 [13]. Participants were randomly recruited from public places, such as parks, malls, cafés, restaurants, exhibitions, and universities. Each participant was informed of their right to privacy and freedom to leave the study at any moment without incurring any obligations to the research team or its objectives. Written informed consent was obtained from all participants. The Regional Research Ethics Committee, Qassim Province (National Committee of Bioethics, No. H-04-Q-001) granted ethics approval for study implementation (registration number: 607-45-3142; approval date: September 20, 2023) and publication (registration number: 607-45-3142; approval date: August 20, 2025).

This study included 1,441 participants residing in the Qassim region. Individuals under the age of 18 years (n = 200) were excluded to examine the quality of life and sleep conditions of the overall adult population; no other exclusion criteria were applied. The participants completed a structured questionnaire survey divided into three sections: sociodemographic and health-related details, Arabic version of the Pittsburgh Sleep Quality Index (PSQI), and the SF-12 survey [14,15]. To improve content validity, a pretest was conducted with 150 eligible participants. The reliability of the scales was confirmed through Cronbach’s alpha, with a coefficient of 0.752 yielded for the overall questionnaire and all domains meeting the standard reliability thresholds.

The Arabic PSQI evaluated seven sleep-related dimensions: sleep latency, habitual sleep efficiency, subjective sleep quality, sleep medication use, sleep duration, sleep disturbances, and daytime dysfunction. The 19-item questionnaire used a mix of 5-point Likert scales and open-ended responses, with component scores ranging from 0 to 3. A global score (range: 0–21) was derived by summing the scores for these dimensions, with higher scores indicating poorer sleep quality (a cutoff of ≥5 defined poor sleep). An alternative three-factor PSQI model was also applied: perceived sleep quality (subjective sleep quality, sleep latency, medication use), daily disturbances (daytime dysfunction, sleep disturbances), and sleep efficiency (habitual efficiency, sleep duration). Both scoring methods were used to obtain comprehensive sleep data.

The SF-12 assesses eight health domains grouped into two summary scores: Physical Component Score (PCS) accounting for physical function, pain, and general health; and mental component score (MCS) accounting for emotional well-being, social function, and vitality. The scores were standardized (mean = 50, SD = 10), with higher values reflecting a better quality of life.

All statistical analyses were performed using IBM SPSS Statistics version 26 (Armonk, NY, USA). Continuous variables are expressed as mean ± standard deviation, while categorical data are presented as frequencies and percentages. Independent-sample t-tests were used to assess the association between sleep quality and quality of life. Pearson’s correlation analysis was performed to examine the bivariate relationships between questionnaire scores. Hierarchical multiple regression was used to evaluate the influence of key factors on HRQoL. Assumptions of normality were verified using Shapiro-Wilk and Kolmogorov-Smirnov tests, and multicollinearity was checked to ensure model validity. Statistical significance was set at p < 0.05, with moderate significance defined as a p-value of <0.01 and strong significance as a p-value of <0.001.

Hierarchical multiple regression analysis was performed in two steps. Step I adjusted for sociodemographic factors (age, sex, nationality, occupation, chronic illness, marital status, and body mass index [BMI]), and Step II included the global PSQI score to examine its association with the SF-12 outcomes (PCS and MCS). Separate regressions were run for each SF-12 component with PSQI scores as predictors and PCS/MCS as dependent variables. Consequently, two hierarchical regression sessions were conducted: one for the MCS and another for PCS. Every regression model was examined for multicollinearity by using the variance inflation factor (VIF), and all categorical variables were dummy-coded. For every variable in the models, the VIF was less than 2. Furthermore, scatter plots of the residuals and predicted values, as well as normal predicted probability plots, were analyzed to verify the homoscedasticity and normal distribution. Every model satisfied the related assumptions. The R2 and adjusted R2 values are presented, along with the regression coefficients and 95% Cis; α = 0.05 was chosen as the two-sided significance level.

## Results

Overall, 1,241 participants completed the survey; 64.5% were female and 94.8% were Saudi nationals. The study population consisted predominantly of young adults, with 43% falling within the 18–20 years age range. Most participants were unmarried (79%) and students (64.8%). Chronic diseases were present in 13.9% of the study population, and 50.9% had a normal BMI (Table 1).

**Table 1:** Participants’ socio-demographic characteristics and health-related characteristics (n = 1,241).

Study variables	N (%)
**Age group, years**	
18–20	534 (43.0)
21–25	370 (29.8)
26–35	171 (15.4)
> 35	146 (11.8)
**Sex**	
Male	441 (35.5)
Female	800 (64.5)
**Nationality**	
Saudi	1,177 (94.8)
Non-Saudi	64 (05.2)
**Marital status**	
Single	980 (79.0)
Married	224 (18.0)
Divorced	23 (01.9)
Widowed	14 (01.1)
**Occupation**	
Student	804 (64.8)
Unemployed	187 (15.1)
Office job	112 (09.0)
Non-office job	89 (07.2)
Freelance job	49 (03.9)
**Presence of a chronic disease**	
Yes	173 (13.9)
No	1,068 (86.1)
**BMI category, kg/m** ^**2**^	
Underweight (<18.5)	190 (15.3)
Normal (18.5–24.9)	632 (50.9)
Overweight (25–29.9)	258 (20.8)
Obesity class I (30–34.9)	102 (08.2)
Obesity class II (35–39.9)	40 (03.2)
Obesity class III (≥40)	19 (01.5)

BMI, body mass index.

**Fig 1:**
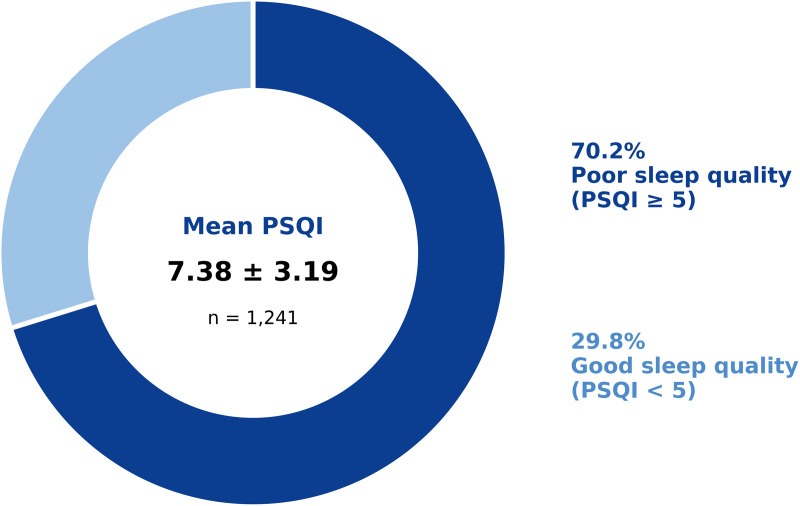
Sleep quality among participants (n = 1241). PQSI, Pittsburgh Sleep Quality Index.

Regarding sleep patterns, the average global PSQI was 7.38 (±3.19), indicating generally poor sleep quality among participants, with a significant proportion (871, 70.2%) classified as having poor sleep quality, while 29.8% (370) reported good sleep quality. The mean scores for specific sleep components were as follows: sleep latency, 1.51 h; sleep duration, 1.44 h; sleep disturbances, 1.26 h; daytime dysfunction, 1.22 h; subjective sleep quality, 1.15 h; habitual sleep efficiency, 0.49 h; and sleep medication use, 0.3 h (Fig 1).

The total mean PCS obtained from the SF-12 survey was 42.6 ± 7.98. The PCS scores were as follows: physical functioning, 0.99; role limitations due to physical health, 0.79; bodily pain, 0.98; and general health perception, 0.63. The total mean MCS was 38.9 ± 8.03, with the mean scores for the mental health, social functioning, role-emotional, and vitality being 1.52, 0.86, 1.09, and 0.87, respectively (Table 2).

**Table 2:** Quality of life among participants (n = 1241).

SF-12 survey Domains	Mean±SD
Physical Component Score (PCS)	42.6 ± 7.98
Mental Component Score (MCS)	38.9 ± 8.03

SF-12, Short Form-12; SD, standard deviation.

Notably, individuals with good sleep quality had considerably greater mental health-related quality of life (as judged by the MCS, p < 0.001; Table 3).

**Table 3:** Differences between sleep quality and quality of life (n = 1,241).

SF-12 survey domains	Level of sleep quality		Effect size (95% CI)	p-value ^§^
	Poor Mean ± SD	Good Mean ± SD		
**Physical Component Score (PCS)**	42.8 ± 7.97	42.3 ± 7.97	0.52 (−0.44 to –1.49)	0.533
**Mental Component Score (MCS)**	38.3 ± 7.97	40.7 ± 7.92	−2.47 (−3.44 to −1.50)	<0.001 ***

§ The p-values are calculated using the independent sample t-test.

* Significant at p < 0.05.

** Significant at p < 0.01.

*** Significant at p < 0.001.

CI, confidence interval; SD, standard deviation; SF-12, Short Form-12.

Marital status (ß = −2.342; p < 0.05) and chronic disease (ß = 2.445; p < 0.05) were significantly associated with physical health-related quality of life, according to the step 1 regression model (R2 = 0.026; F = 5.774; p < 0.001). Hierarchical regression revealed that chronic disease (β = 0.078, p < 0.01), marital status (β = −0.090, p < 0.05), and perceived sleep quality (β = 0.073, p < 0.05) were significant predictors of physical health-related quality of life. However, the standardized coefficients were small, indicating modest individual contributions to the variance in physical health outcomes. The inclusion of PSQI components in Step 2 substantially improved the model (ΔR² = 0.034, F = 5.401, p < 0.001) (Table 4).

**Table 4:** Hierarchical multiple regression model with physical health-related quality of life (n = 1,241).

Variables	Step 1			Step 2		
	Coefficient (SE)	t	Beta	Coefficient (SE)	T	Beta
**Step 1**						
Age	−0.032 (0.038)	−0.836	−0.039	−0.025 (0.038)	−0.670	−0.031
Sex	0.773 (0.496)	1.560	0.046	0.877 (0.499)	1.758	0.053
Nationality	−0.799 (1.019)	−0.784	−0.022	−0.770 (1.022)	−0.754	−0.021
Marital status	−1.437 (0.614)	−2.342 *	−0.098	−1.321 (0.612)	−2.157 *	−0.090
Occupation	−0.158 (0.248)	−0.636	−0.023	−0.152 (0.248)	−0.613	−0.022
Chronic disease	1.681 (0.687)	2.445 *	0.073	1.796 (0.691)	2.599 **	0.078
BMI	0.157 (0.240)	0.653	0.020	0.137 (0.240)	0.571	0.018
**Step 2**						
Sleep efficiency				0.474 (0.252)	1.885	0.057
Perceived sleep quality				0.669 (0.281)	2.384 *	0.073
Daytime disturbances				−0.167 (0.398)	−0.419	−0.013
**Summary statistics**						
Model F	5.774 ***			5.401 ***		
R2	0.032			0.042		
Adjusted R^2^	0.026			0.034		
R^2^ change F	5.774 ***			5.401 ***		

BMI, body mass index; SE, standard error.

* Significant at p < 0.05.

** Significant at p < 0.01.

*** Significant at p < 0.001.

The initial analysis (Step 1) showed significant associations between mental health and age (β = 0.113, p < 0.05) and sex (β = −0.146, p < 0.001). After incorporating sleep quality domains (Step 2), sex (β = −0.149, p < 0.001), BMI (β = −0.069, p < 0.05), and perceived sleep quality (β = −0.199, p < 0.01) remained significant predictors of mental health-related quality of life. The standardized coefficients indicated generally small effect sizes, with perceived sleep quality showing the strongest, albeit modest, association with sleep quality. The enhanced model explained substantially more variance (ΔR² = 0.094, F = 13.833, p < 0.001) (Table 5).

**Table 5:** Hierarchical multiple regression model with mental health-related quality of life (n = 1241).

Variables	Step 1			Step 2		
	Coefficient (SE)	t	Beta	Coefficient (SE)	t	Beta
**Step 1**						
Age	0.094 (0.038)	2.462 *	0.113	0.071 (0.037)	1.912	0.085
Sex	−2.456 (0.496)	−4.953 ***	−0.146	−2.506 (0.486)	−5.152 ***	−0.149
Nationality	−0.336 (1.019)	−0.330	−0.009	0.114 (0.997)	0.115	0.003
Marital status	1.172 (0.614)	1.909	0.079	0.896 (0.597)	1.501	0.060
Occupation	0.086 (0.248)	0.348	0.012	0.014 (0.241)	0.058	0.002
Chronic disease	0.043 (0.688)	0.063	0.002	−0.570 (0.674)	−0.845	−0.025
BMI	−0.603 (0.240)	−2.507	−0.078	−0.531 (0.234)	−2.272 *	−0.069
**Step 2**						
Sleep efficiency				−0.268 (0.245)	−1.092	−0.032
Perceived sleep quality				−1.831 (0.274)	−6.692 **	−0.199
Daytime disturbances				−0.968 (0.388)	−2.492	−0.073
**Summary statistics**						
Model F	8.164 ***			13.833 ***		
R2	0.044			0.101		
Adjusted R^2^	0.039			0.094		
R^2^ change F	8.164 ***			13.833 ***		

BMI, body mass index; SE, standard error.

* Significant at p < 0.05.

** Significant at p < 0.01.

*** Significant at p < 0.001.

Post-hoc power analyses were performed to evaluate the adequacy of the sample size for the primary inferential analyses. For the comparison of quality-of-life scores between participants with poor and good sleep quality, the observed difference in mental health-related quality of life (MCS) corresponded to a small-to-moderate effect size (Cohen’s d = 0.30), yielding an achieved power of 99.8% at a two-sided significance level of 0.05. In contrast, the difference in physical health-related quality of life (PCS) corresponded to a trivial effect size (Cohen’s d = 0.06), resulting in an achieved power of 17.3%.Regarding the hierarchical multiple regression analyses, post hoc power calculations based on the observed coefficients of determination demonstrated excellent statistical power. Both the PCS and MCS models (R² = 0.032 and 0.042; R² = 0.044 and 0.101, respectively) achieved powers exceeding 99.9%. Overall, the sample size of 1,241 participants was more than adequate for detecting the observed effects, except for the trivial difference observed in PCS between the sleep quality groups.

## Discussion

The current study showed that the overall mean PSQI was 7.38, with 70.2% of the participants experiencing poor sleep quality. The mean MCS and PCS scores for the SF-12 survey were 38 ± 8.03 and 42.6 ± 7.98, respectively. After controlling for demographic characteristics, chronic disease, and BMI, sleep-related variables significantly predicted both physical and mental health-related quality of life. The analysis showed that sleep-related factors had a substantially larger predictive contribution toward mental health (ΔR2 = 5.7%) than toward physical health (ΔR2 = 1.0%).

According to the current study, 70.2% of the people in the Qassim region of Saudi Arabia had poor sleep quality, with a mean PSQI score of 7.38. This observed prevalence in Saudi Arabia exceeds that reported in Brazil (65.5%), Germany (35.9%), Austria (32.1%), Hong Kong (39.4%), and Shanghai (8.3%) [16–18].

This study supports and expands the findings of other studies, indicating a potential relationship between quality of life and sleep quality. Significant associations were observed between most SF-12 survey components and sleep quality. Currently, health-related quality-of-life research lacks standardized thresholds for clinically meaningful changes. However, previous research in diabetic populations has shown that even a single-point decline in SF-36 measures corresponds to an increased mortality risk (up to 9%) and a higher likelihood of work disability (12%) [19].

The concept of the minimally important difference (MID) has frequently been the subject of health-related quality-of-life research to establish standards for interpreting changes. For MIDs, 2–4 points are generally considered clinically significant [20]. A common MID criterion of a 5-point difference in the SF-12 survey summary scores has also been proposed to indicate significance [21,22]. Consequently, interpretation becomes more challenging when the observed difference is small but significant [23]. Therefore, the current results imply clinically significant variations in quality of life between those who had poor sleep quality and those who did not.

After controlling for sex and BMI, this investigation revealed significant relationships between sleep quality and mental health outcomes. Similarly, notable associations emerged between sleep quality and physical health dimensions after accounting for chronic diseases and marital status. These findings corroborate the existing literature while underscoring the importance of considering socioeconomic status, cultural influences, health-related covariates, and other established quality-of-life determinants [24,25]. Although the observed effect sizes were modest, their potential clinical relevance was strengthened by comprehensive adjustment for confounders in the regression models.

According to recent research, BMI is significantly correlated with aspects of quality of life related to mental health. A previous study found a correlation between BMI and several mental aspects of HRQoL in individuals with obstructive sleep apnea [26]. Previous studies have shown strong associations between BMI and components of physical health-related quality of life [27–29].

Previous studies suggested that BMI can affect sleep [25,30,31]. However, hierarchical regression models showed that none of the variables (chronic illness, BMI, and demographics) had a VIF greater than 2 and that adding sleep variables one at a time significantly improved prediction in every model. Thus, the current findings demonstrate that subjective sleep problems are universally linked to a declining HRQoL. Although sleep disorders are a major global issue, the prevalence of sleep disorders and the degree to which HRQoL is affected by sleep are underreported in the developing world. This finding suggests that more attention should be paid to sleep care.

Additionally, studies have shown significant associations between obstructive sleep apnea, insomnia, daytime drowsiness, and HRQoL. Only severe sleep problems showed these relationships, not all SF-12 survey measures [32–34]. Some general sociological characteristics have been hypothesized to increase the likelihood of psychosocial instability by influencing the relationship between sleep deprivation and all facets of HRQoL.

This theory is consistent with the research showing that poor socioeconomic status has detrimental effects on sleep and health [35,36]. Some of these findings imply that sleep may moderate the relationship between ill health and socioeconomic status [35]. Numerous research projects have also shown a negative correlation between HRQoL and socioeconomic status [37,38]. Although depression and socioeconomic status might be factors influencing poor sleep, earlier research has found that sleep issues are independent indicators of lower quality of life. How much of the gradient in the impact of sleep on HRQoL can be explained by economic sleep patterns remains unknown. Given that economic position is the most important predictor of poor sleep, this underscores the need to investigate sleep problems and their impact on HRQoL in individuals with low socioeconomic status.

This study had some limitations. The cross-sectional design prevented the establishment of a causal relationship between sleep quality and HRQoL. The use of convenience sampling also restricted the generalizability of the results to broader populations. Additionally, sleep data were self-reported via questionnaires rather than objective measures, which potentially introduced bias. While some SF-12 and PSQI items appeared similar, this concern was mitigated using the PSQI global score, a composite measure of seven sleep components. This study did not account for all potential lifestyle, health, and mental health factors that could influence HRQoL. Confounding variables related to physical and mental health conditions, often associated with sleep disturbances, may have affected the outcomes, although chronic disease status was controlled to reduce this bias. Notably, underlying health issues could worsen sleep problems and further reduce HRQoL. Although the specific causes of sleep disruption were not examined, the adjusted analysis (accounting for demographics, BMI, and chronic conditions) revealed clinically meaningful associations between sleep disorders and HRQoL, supporting a likely bidirectional relationship.

This study also has several notable strengths. Standardized, widely accepted measures were used to collect data. Furthermore, most participants did not receive any kind of therapy for sleep disturbance. Moreover, to strengthen the relationships found, the association between sleep and HRQoL was further evaluated across sleep quality in the same population sample, which comprised participants with and without sleep disturbance, as well as good and poor sleepers.

## Conclusions

This study showed a high prevalence of poor sleep quality (70.2%) in the general population of Qassim region. Poor sleep quality was associated with lower mental health-related quality of life and remains an important predictor of mental well-being after adjusting for demographic and health-related factors. In contrast, the associations with physical health-related quality of life were weaker, and the individual effect sizes of the significant predictors were generally small, suggesting limited practical significance despite statistical significance.

From a public health perspective, these findings support the integration of sleep health promotion into population health strategies. Specific actions may include incorporating sleep quality screening into primary healthcare services, implementing community-based sleep hygiene education programs, and promoting healthy sleep behaviors through public health campaigns. Future longitudinal studies are warranted to confirm these associations and investigate additional mediating and moderating factors that influence the relationship between sleep quality and HRQoL.
